# Analysis of Bacteroides fragilis Clinical Strains Isolated in Kazakhstan

**DOI:** 10.1128/MRA.01311-20

**Published:** 2021-02-04

**Authors:** Elena Zholdybayeva, Saniya Kozhahmetova, Pavel Tarlykov, Sabina Atavliyeva, Kymbat Mukhtarova, Tleuli Syzdykov, Ruslan Khasenov, Alexsandr Shevtsov, Asylulan Amirgazin, Asset Daniyarov, Yerlan Ramankulov

**Affiliations:** a RSE National Center for Biotechnology, Nur-Sultan, Republic of Kazakhstan; b SOE No. 1 City Hospital, Nur-Sultan, Republic of Kazakhstan; c SOE No. 2 Multidisciplinary Regional Hospital, Nur-Sultan, Republic of Kazakhstan; d Laboratory of Bioinformatics and Systems Biology, Center for Life Sciences, National Laboratory Astana, Nazarbayev University, Nur-Sultan, Republic of Kazakhstan; e School of Science and Technology, Nazarbayev University, Nur-Sultan, Republic of Kazakhstan; University of Maryland School of Medicine

## Abstract

Our aim was to study the nucleotide sequences of 9 previously undescribed strains of B. fragilis collected from patients with intra-abdominal diseases at city hospitals in Nur-Sultan, Kazakhstan.

## ANNOUNCEMENT

Bacteroides fragilis is a commensal bacterium that is found in the intestines of most people and that can become an opportunistic pathogen. Amounting to only about 0.1 to 0.5% of the total bacterial mass of the intestine, B. fragilis is the most frequently isolated anaerobe from clinical samples obtained from deep intra-abdominal abscesses, purulent skin infections, soft tissue infections (1, 2), diarrhea, and colorectal cancer (3).

B. fragilis strain no. 4 to 12 were isolated from clinical samples of patients diagnosed with acute peritonitis receiving treatment at City Hospitals No. 1 and No. 2 and Regional General Hospital No. 2. Informed consent and questionnaires were approved by the local ethics committee of the RSE National Center for Biotechnology of the Ministry of Education and Science of the Republic of Kazakhstan (extract from protocol no. 4 of 29 August 2017).

**FIG 1:**
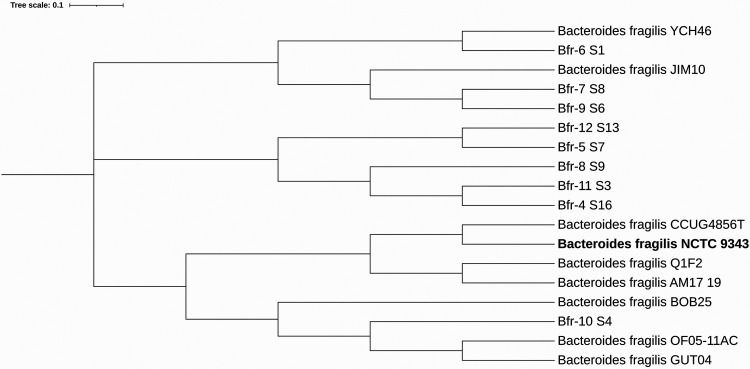
Phylogram of available Bacteroides fragilis genomes based on the concatenated alignment of the high-quality SNPs using CSI Phylogeny v1.4. The following available genomes were retrieved from GenBank (ncbi.nlm.nih.gov/genome/): Bacteroides fragilis strain JIM10 (GenBank accession no. CM004507.1), Bacteroides fragilis strain YCH46 (NC_006347.1), Bacteroides fragilis strain CCUG4856T (CP036555), Bacteroides fragilis strain Q1F2 (NZ_CP018937.1), Bacteroides fragilis strain AM17-19 (NZ_QRJX00000000.1), Bacteroides fragilis strain BOB25 (CP011073.1), Bacteroides fragilis strain OF05-11AC (NZ_QSWE00000000.1), and Bacteroides fragilis strain GUT04 (CP043610.1). The reference genome, Bacteroides fragilis NCTC 9343 (NC_003228.3), is indicated in bold.

Samples were collected from the drainage wounds by use of swabs with subsequent immersion of probes into tubes containing Amies medium. After that, samples were cultivated on *Bacteroides* bile esculin agar (BBE; Conda) at 37°C for 72 h under anaerobic conditions (an aerostat, gas pack). Isolates were identified using matrix-assisted laser desorption ionization–time of flight mass spectrometry (MALDI-TOF MS; Bruker Daltonics, Bremen, Germany). Bacterial colonies growing on solid medium were removed with a sterile plastic tip and resuspended in 1,000 μl of Tris-EDTA (TE) buffer. Total DNA was extracted from all strains using the cetyltrimethylammonium bromide (CTAB) method (4). Then, 150 ng of the total genomic DNA from each isolate of B. fragilis was used for sequencing. Library preparation and Illumina MiSeq sequencing were performed using the Nextera DNA Flex library prep kit and a MiSeq reagent kit v3 with 300-bp paired-end reads (600 cycles) according to the manufacturer’s instructions. Quality assessment of the sequencing data (in FASTQ format) was done using FastQC v0.11.15 (5), followed by trimming of adapters and low-quality bases with a Phred quality score of less than 20 using Trimmomatic (6). Genomes were assembled using the SPAdes assembler v3.13.2 using a k-mer length of 127 with the “--careful” mode (7). Comparative phylogenetic analysis was performed with the CSI Phylogeny v1.4 tool from whole-genome sequences using the following parameters: minimum depth, 80×; minimum relative depth, 100%; minimum distance between single-nucleotide polymorphisms (SNPs), 1,000 bp; minimum SNP quality, 500; minimum read mapping quality, 500; and minimums Z-score, 3.29 (8). We discarded sequences with low quality, i.e., ambiguous bases. Figure 1 was created using iTOL v4 (9). B. fragilis strain NCTC 9343 (GenBank accession no. NC_003228.3) was used as a reference sequence. Genome annotation was performed using PGAP v4.11 (10). Default parameters were used for all software.

Data from the whole-genome sequencing of the 9 clinical B. fragilis strains are presented in Table 1.

**TABLE 1:** Genome characteristics of 9 B. fragilis strains

Strain no.	Strain name	Genome size (bp)	Genome coverage (×)	No. of contigs	Total no. of reads	Avg read length (bp)	*N*_50_ (bp)	G+C content (%)	Accession no./SRA no.
4	Bfr-4	4,920,846	20.0	128	1,724,380	192	69, 701	43.41	JACEFH000000000/SRX9403175
5	Bfr-5	5,219,927	38.565	77	1,555,229	221	181, 129	43.36	JACENG000000000/SRX9403182
6	Bfr-6	5,259,406	49.394	41	1,398,871	231	378, 644	43.33	JACFSS000000000/SRX9404153
7	Bfr-7	5,175,039	19.59	28	736,748	273	379, 532	43.25	JACFST000000000/SRX9404154
8	Bfr-8	5,243,270	64.17	40	1,258,909	242	311, 507	43.24	JACFSU000000000/SRX9404155
9	Bfr-9	5,125,231	29.757	53	1,501,107	218	290, 748	43.45	JACFSV000000000/SRX9404156
10	Bfr-10	5,242,965	31.158	36	1,511,363	226	397, 964	43.12	JACFSW000000000/SRX9404157
11	Bfr-11	5,236,934	33.68	32	1,386,003	245	407, 555	43.4	JACFSX000000000/SRX9404158
12	Bfr-12	5,321,320	31.143	92	1,272,842	229	182, 180	43.39	JACFSY000000000/SRX9404159

Results of the phylogenetic analysis are shown in Fig. 1. B. fragilis Bfr-10 is located farther from all the strains studied.

### Data availability.

The 9 whole-genome shotgun projects have been deposited at DDBJ/ENA/GenBank under the accession numbers listed in Table 1.
